# Addressing stigma within the dissemination of research products to improve quality of care for pregnant and parenting people affected by substance use disorder

**DOI:** 10.3389/fpsyt.2023.1199661

**Published:** 2023-06-07

**Authors:** Megan Lipsett, Katie Wyant-Stein, Simone Mendes, Estelle Berger, Elliot T. Berkman, Mishka Terplan, Camille C. Cioffi

**Affiliations:** ^1^Department of Psychology, Center for Translational Neuroscience, University of Oregon, Eugene, OR, United States; ^2^Diamond Lab, Department of Psychology, University of Utah, Salt Lake City, UT, United States; ^3^Friends Research Institute, Baltimore, MD, United States; ^4^Prevention Science Institute, University of Oregon, Eugene, OR, United States

**Keywords:** stigma, substance use disorder, dissemination, perinatal, harm reduction, parenting, treatment accessibility, health equity

## Abstract

Substance use disorders are a common and treatable condition among pregnant and parenting people. Social, self, and structural stigma experienced by this group represent a barrier to harm reduction, treatment utilization, and quality of care. We examine features of research dissemination that may generate or uphold stigmatization at every level for pregnant and parenting individuals affected by substance use disorder and their children. We explore stigma reduction practices within the research community that can increase uptake of evidence-based treatment programs and prevent potential harm related to substance use in pregnant and parenting people. The strategies we propose include: (1) address researcher stereotypes, prejudice, and misconceptions about pregnant and parenting people with substance use disorder; (2) engage in interdisciplinary and transdisciplinary collaborations that engage with researchers who have lived experience in substance use; (3) use community-based approaches and engage community partners, (4) address stigmatizing language in science communication; (5) provide contextualizing information about the social and environmental factors that influence substance use among pregnant and parenting people; and (6) advocate for stigma-reducing policies in research articles and other scholarly products.

## Introduction

More than 40 million Americans struggled with substance use disorders (SUD) in 2020 (1), many of whom are statistically likely to be pregnant or parenting (2, 3). It is difficult to estimate the national prevalence of SUD among pregnant and parenting people (e.g., due to the illegal nature of some substance use and lack of coordinated tracking across treatment facilities), but the prevalence of SUD among this group seems to be increasing (4). It has been estimated that 1 in 8 children live with a parent with a SUD (3).

The efficacy of evidence-based therapeutic interventions for SUD has now been established, with benefits across individuals, families, and society [e.g., (5, 6)]. Yet, only 1 in 5 people with SUD report receiving the treatment they need. Stigma has been named as a barrier to receiving care (1). The National Institute on Drug Abuse (NIDA) has identified stigma reduction as a major priority, emphasizing that stigma inhibits the implementation and adoption of effective treatments and harm-reduction approaches [e.g., medications for opioid use disorder and other addictions, syringe service programs, and fentanyl testing strips to avoid unintentional fentanyl exposure; (7)]. Social, self, and structural stigma toward individuals who use substances, which is notably higher than stigma toward those with mental illness broadly (8), is an important driver of low uptake of and adherence to these programs. This may be especially true for pregnant and parenting people with SUD who may experience greater stigma based on their pregnancy and parenting status (9). Despite growing knowledge of the genetic and social determinants of SUD, stigma toward pregnant and parenting people who use substances remains a barrier to accessing care and a significant public health concern.

There is a need to promote stigma reduction which may also reduce substance-related harm to parents and their children. While stigma exists at many levels of society, this article explores opportunities for the research community to mitigate substance-related stigma toward pregnant and parenting people through research dissemination of scholarly and non-scholarly products. These research products include journal articles, conference presentations, and community-facing information on findings. How researchers articulate and contextualize their research findings [e.g., through the rhetoric of maternal unfitness; (10)] can have consequences for intervention uptake, public perception, policy, and the way practitioners perceive, communicate, and treat pregnant and parenting people with SUD. Researchers can examine the existence of stigma within their work as part of a larger effort to alleviate the adverse effects of both stigma itself and the impacts of stigma on accessing health services.

We present strategies within a unifying framework to reduce stigmatization of pregnant and parenting individuals who use substances in the dissemination of research findings. The strategies we propose include: (1) address researcher stereotypes, prejudice, and misconceptions about pregnant and parenting people with SUD; (2) engage in interdisciplinary and transdisciplinary collaborations that engage with researchers who have lived experience in substance use; (3) use community-based approaches and engage community partners, (4) address stigmatizing language in science communication; (5) provide contextualizing information about the social and environmental factors that influence substance use among pregnant and parenting people; and (6) advocate for stigma-reducing policies in research articles and other scholarly products.

### Impacts of social, self, and structural stigma surrounding substance use disorder on pregnant and parenting people

Despite the prevalence of pregnant and parenting people with SUD, only about 9% of those with SUD receive any kind of treatment (11). While these treatment rates are driven by multiple structural factors (e.g., limited availability of treatment centers, inability to access or afford care, and limited screening for SUD in medical visits), fear of shame and stigmatization in seeking care remains a key determinant of treatment engagement (12–14). Indeed, stigma has been proposed as a key barrier to treatment utilization for those with SUD (1)–and SUD is the most globally stigmatized health condition according to the World Health Organization (15). Researchers first need to understand the impact of stigma on this population.

Stigma is embedded at multiple layers of social interaction. Pregnant and parenting people who use substances may experience compounding social, structural, and self- stigmatization. Social stereotypes manifest behaviorally (e.g., by social distancing or discrimination) and, ultimately, hinder care delivery and undermine treatment access (16). Stigma at every level increases distress, social isolation, and diminished access to resources (17). Stigma also influences the progression from substance use to the development of a SUD, undermines SUD treatment efforts, and drives health inequities across the life course (18). Stigmatization of opioid use, for instance, has hindered the national response to the opioid crisis in the United States by reducing public support for beneficial programs, such as the uptake of effective harm reduction strategies and evidence-based treatment [e.g., medication for opioid use disorder; (16)].

*Social stigma* arises from stereotypes about people who use substances in general, including harmful narratives that people who use substances are dangerous or purposefully choose not to abide by moral societal standards. When health professionals endorse stereotype perceptions (e.g., assigning poor motivation to patients) and display diminished empathy, this impacts patient empowerment, quality of care, and the type of treatment that parents with SUD receive (8, 19). Disregarding patient autonomy and engaging in nonbeneficent care policies occurs disproportionately among care providers when treating people who use substances (16). The suboptimal medical care that results from stigma also occurs with respect to children of parents who use substances, including when medical conditions are assumed to be connected to substance exposure rather than their root cause (19–23). Ultimately, stigma impacts appropriate healthcare provision via barriers to health-seeking behavior, engagement in structures of care, and treatment adherence (24). Further, there are racial disparities in access to treatment for SUD (25), emphasizing that pregnant and parenting people who use substances and who have a minoritized racial identity are at risk of racial discrimination and greater stigmatization. Degree of social stigma can vary based on substance used. This form of social stigma is more often related to racialized narratives than to pharmacological impacts of the substances themselves (26). Indeed, more severe substance-specific social stereotypes have been weaponized to attack the parental fitness of minoritized groups (27). Conversely, substances commonly used by White, middle class individuals (e.g., alcohol and cannabis) tend to have less severe societal stigma (28). As such, it is important to consider racialized narratives that underly individual ideologies about the impacts of use.

Stereotypes about pregnant and parenting people with SUD, specifically, are often related to the potential of (1) prenatal substance use to cause fetal harm and (2) parental substance use to cause harm to the child. When examining these potential risks, social narratives commonly link substance use with “maternal unfitness” and focus on parental deficits, particularly when parents are compared to hegemonic ideals of parenthood (29). While it is critical to prevent and address potential harms associated with substance use during pregnancy, the scientific evidence of actual meaningful consequences of exposure does not support the extent to which SUD has been linked to the unfitness of the birthing person to parent (10, 30). Instead, the narrative of parental unfitness undermines the importance of the parent -baby bond and often leads to depriving both mother and baby of the benefit of embeddedness in a supportive environment (30).

Focusing on parental deficits implies that parents who struggle with substance use are wholly unable to provide adequate nurturing to their children (31), despite the evidence of treatment and harm reduction approaches demonstrating that there are ways to use substances and still have strong parenting skills. For instance, our Center on Parenting and Opioids provides parenting resources to support parents to reduce substance use if that is their goal, reduce potential harm to self or child, and prepare for safe and successful parenting [e.g., obtaining additional supportive care when planning to use, not sleeping in the same bed with children, avoiding putting children around water, and keeping substances in medication boxes and away from children’s access; (32)]. This and other treatment and harm reduction approaches (e.g., programs like HomeSafe in New Jersey) prioritize meeting the primary needs of the whole family by providing wrap-around services rather than penalizing the parent for use of substances (33).

Pregnant and parenting people with SUD are also vulnerable to *structural stigma*, wherein social stigma becomes embedded in cultural norms, laws, and the policies and procedures of institutions, restricting their rights and opportunities (16, 34). For instance, drug use in pregnancy is codified as child abuse and results in parenting individuals with a record of child abuse. Structural stigma results in limited access to housing, work opportunities, and medical and behavioral treatment (35). The narrative of “maternal unfitness” has led to government interventions and punitive actions against pregnant people, such as arrest, detention, loss of parental custody, or “lock-in” programs, in which individuals are restricted to only obtaining substances from a single pharmacy (10, 36, 37). Though designed to protect fetal health, these responses fail to consider scientific evidence related to individual values and motivations, successful treatment of SUD, and structural factors that undermine treatment utilization for both SUD and obstetrical care (10). In fact, punitive responses can exacerbate the issue, including predicting increased likelihood of infant Neonatal Abstinence Syndrome [NAS; (38, 39)].

Punitive policies are centralized around criminalization, leading to high rates of prosecution and incarceration, family separation (40–42), and fewer resources to support those who are affected by substance use [e.g., restrictions on treatment, limited access to overdose prevention sites, and fewer social services generally; (43)]. Punitive approaches further increase the likelihood that pregnant people (21), will avoid accessing healthcare, ultimately widening health inequities (44–46). The impact of stigma on healthcare utilization may be further compounded for parents facing housing instability or houselessness, who often do not seek out supportive services for fear of being separated from their children.

Stigma may impede the advancement of evidence-based healthcare delivery policies (8) and policies oriented toward public health, which are designed to support individuals through expanded services for prevention and treatment (37). Public health-oriented policies, such as the expansion of Medicaid insurance to cover prescription opioid treatment or laws that ensure that those seeking help for overdose are protected from criminal charges, aim to address substance use without inhibiting healthcare access (37). Unlike punitive approaches, public health approaches are grounded in the understanding of SUD as a chronic, but treatable, condition. In fact, many people successfully manage SUD (47) and there are many examples of pregnant and parenting people with SUD that demonstrate hope, resilience, and restoration (16).

*Self-stigma* occurs when pregnant and parenting individuals internalize negative societal beliefs and sentiments (48). Internalized narratives become predictive as individuals with SUD anticipate stigmatizing and judgmental treatment when interacting with providers and healthcare systems; ultimately leading to poorer health outcomes (16, 49) and retention and follow-up care challenges (50). Impacts of self-stigma on psychological well-being, self-efficacy and resultant treatment outcomes for those with substance use disorders are well-documented (51–54). The downstream mental health consequences of self-stigma may itself be a determinant for continued substance use. For instance, Khantzian’s (55) *Self-medication Hypothesis* posits the misuse of substances as a self-regulation strategy to manage difficult life experiences, including stigma-related social anxiety and experiences of racial discrimination (38, 56, 57). Interventions that target experiences internalized stigma such as Acceptance and Commitment Therapy, are effective for reducing substance use and increasing treatment attendance (58, 59).

Overall, stigma harms pregnant and parenting individuals with SUD and their children by preventing access to necessary and vital services, contributing to internalized social devaluation, psychological distress, and underutilization of treatment (34, 48). These psychosocial and economic impacts further perpetuate challenges to parenting, impacting the families and children of parents with SUD. Critically, choices made within the research process such as language used to describe pregnant and parenting people who use substances and lack of inclusion of the role of stigma as a confounder to measured outcomes may perpetuate stigmatization and resulting discrimination. Examining opportunities to prevent the downstream impacts of research approaches that lead to stigma is an important step in creating supportive environments for pregnant and parenting people with SUD to ultimately improve health outcomes for this vulnerable population.

### The role of research in shaping public perceptions, treatment utilization, and policy

Stigmatization that is created or upheld within research products may impact care experiences for pregnant and parenting individuals affected by SUD and their children by contributing to social, structural, and self-stigma. Stigma may arise in how research is conducted, shared, and interpreted. In many ways, effectively destigmatizing research starts even before the data are collected; it begins with working alongside and uplifting the voices of the individuals and communities under examination (60). Stigmatizing beliefs exist within research toward individuals who use substances generally, as described by Stull et al. (61), p. 2:

‘Those of us who have conducted human research have noticed that beliefs about addiction are incorporated into every aspect of it–the framing of questions, the screening criteria for studies, the experimental manipulations used in human laboratory sessions, and the outcome measures used in clinical trials or assessment studies. Most of those beliefs are based on data and are defensible, but often they do not allow for the degree of heterogeneity that we know, from experience, is characteristic of addiction.’

Ultimately, there is a need for a “toolbox” of various strategies and evidence-based interventions that address the multiple levels at which stigma arises [e.g., (17, 62, 63)]. However, to provide timely guidance to researchers who are ready to share findings, the remainder of this paper will focus on strategies to reduce stigma toward pregnant and parenting people with SUD as it relates to the dissemination of research products.

Within dissemination, stigma may arise in how we portray our research, how it is shared, and how it is interpreted; impacting health and well-being experiences for pregnant and parenting individuals affected by substance use disorder. Stigma is a driving factor in the persistent barriers to the equitable and efficient translation of research into clinical practice, public health benefit, and policy change (64). There have been numerous calls for research to be increasingly patient-oriented (65, 66) and sensitive to the social impact of scientific research (67)–and for the creation of guidelines to support researchers in considering the way in which they frame SUD during publication (68). Stigmatizing pregnant and parenting individuals with SUD may prevent evidence-based practices in reaching implementation stages and later translation to community health policies and programs. To the extent that research products inform policy and institutional procedure, these products may inadvertently perpetuate structural stigma. Researchers may lend support to punitive approaches by failing to explore the potential for public health and harm reduction approaches to confer beneficial outcomes for pregnant and parenting people who use substances and their families.

Eliminating the use of stigmatizing narratives within the dissemination of research products is one step toward improving the ways society and care providers interact with pregnant and parenting people with SUD. The unconscious and insidious nature of stigma suggests the need for our active engagement to decrease substance-related stigma toward pregnant and parenting individuals in research dissemination. Using a framework to guide the research process increases impact (69). There are dozens of frameworks of research dissemination and implementation, with much of the guiding content focused on structural aspects of dissemination, such as research planning and measures selection (69). To our knowledge, there are no comprehensive frameworks to reduce stigma within research products related to SUD among pregnant and parenting individuals.

### A framework of strategies to reduce stigma in the dissemination of research findings

We present the following strategies for guiding efforts to reduce stigma within research dissemination for pregnant and parenting people affected by SUD. We draw from related frameworks [e.g., The Health Stigma and Discrimination Framework; (26)], which articulate the process of stigmatization in the context of health broadly, to frame stigma-reduction strategies within research dissemination to reduce harm caused to pregnant and parenting individuals with SUD. We suggest how key components of these frameworks might be integrated into the dissemination of research products for those conducting research involving pregnant and parenting people with SUD. We provide a multi-level approach to consider the various levels that may perpetuate or address stigma (18). These strategies are organized by order of operations when approaching dissemination planning and procedures for research efforts (see Table 1).

**Table 1:** Strategies for reducing stigma toward pregnant and parenting people with SUD within research dissemination.

Address researcher stereotypes, prejudice, and misconceptions about pregnant and parenting people with SUD
Engage in interdisciplinary and transdisciplinary collaborations–emphasizing engagement with researchers with lived experience
Use community-based approaches and engage community partners
Address stigmatizing language in science communication
Provide contextualizing information about the social and environmental factors that influence substance use among pregnant and parenting people
Advocate for stigma-reducing policies in research articles and other scholarly products

### Actionable recommendations

#### Strategy #1: address researcher stereotypes, prejudice, and misconceptions about pregnant and parenting people with SUD

Despite positive intentions within the research community, normative judgments that exist at both conscious and unconscious levels are reflected in scientific endeavors and perpetuate stigma (70). Specifically, generalized assumptions that people with SUD are incapable of providing a supportive caregiving environment are reflected in academic language and are used to justify harmful and unwarranted child welfare reporting practices. Stigmatizing attitudes are known to be most prevalent among those without knowledge of or experience with a stigmatized condition [e.g., (71)]. Without direct experience, for instance, researchers might not have had the opportunity to observe pregnant and parenting people who use substances acting as competent parents or recovering from SUD. Narratives that over-emphasize negative attributes and life circumstances can increase stigmatization (72). Effective stigma reduction strategies, such as providing education on stigma reduction or social contact interventions that focus on sharing experiences or stories of competent parenting or recovery among pregnant and parenting people with SUD, can be incorporated into research dissemination practices. For example, including a narrative account in tandem with quantitative research findings. Additionally, the enhanced stigmatization that is accompanied by the use of certain substances such as heroin (73) and methamphetamine (74) or the modality of use such as injection (75), necessitates the inclusion of parents with specific lived and living expertise to provide their accounts of parenting, particularly as it relates to the pain of removal, the hope of family cohesion, and the successful navigation of parenting when adequate social supports exist.

Evidence of the detrimental impacts of the negative attitudes upheld by health professionals [i.e., diminished communication and hindering the therapeutic alliance for clinical interventions; (76)] suggests that negative attitudes among researchers may have similar negative consequences. Researchers can examine personal biases toward pregnant and parenting people who use substances to mitigate potential harm resulting from research products. Beyond the potential to create negative experiences and outcomes of study participants, impact study adherence, and inhibit researcher awareness about relevant study events, diminished communication can negatively influence our theoretical approaches and interpretation of findings. Like ‘diagnostic overshadowing,’ wherein physical illness symptoms are misattributed to mental illness and result in underdiagnosis (77), our theoretical framings may incorrectly disregard important participant comorbidities or inaccurately assume they are a feature of substance use. The perceptions we hold about the controllability and culpability of having SUD, known as attributional beliefs, may impact the way we frame SUD in our research products. For instance, attributional beliefs may lead to reporting about SUD using a framework based on individualism and personal responsibility (e.g., narratives of “maternal unfitness”) rather than discussing upstream and social causes, which can change social perceptions and ultimately reduce motivation for social and interpersonal responsibility-taking behaviors (78). Meaningful interaction with individuals who have had the direct experience of substance use while pregnant and parenting may reduce biases by reducing anxiety related to those interactions, increasing knowledge about the lived experience of those individuals, and enhancing empathy [e.g., Pettigrew and Tropp’s contact hypothesis; (79)].

Models that recognize the physiological and psychosocial drivers of SUD (e.g., disease models and biopsychosocial models) may minimize the moralization of substance use and therefore reduce stigmatizing beliefs toward pregnant and parenting people who use substances. NIDA promotes the characterization of SUD as a “chronic relapsing brain disorder” that “powerfully compromises executive function circuits that mediate self-control and decision-making” as a method of stigma reduction toward those with SUDs (7). Compared to other models that have a focus on will power or personal characteristics as a driving element of SUD, recognizing the complex biopsychosocial drivers of SUD is both more accurate and more likely to prevent stigma. For example, the disease model asserts that a pregnant person who uses substances during pregnancy can be seen as someone suffering from a chronic disorder, rather than as someone who is choosing to actively harm their developing fetus. However, disease models may also be vulnerable to the process of moralization (80), and require reflection on how assertions of moral responsibility within our theoretical approaches may inform stigma. Biopsychosocial models consider psychological attributes, individual skills, and social and environmental context, which may increase understanding about the internal mechanisms of use within the individual. The integration of these models is important for providing holistic care for pregnant and parenting people with SUD. However, neither of these models fully address social and structural determinants of health, which are key drivers of parent substance use (81, 82).

To the extent that stigma occurs at the unconscious level, it is crucial to proactively support stigma reduction efforts by increasing awareness of social, self, and structural stigma. Specifically, we propose developing a clear understanding of stigma and its effects, becoming aware of and responsive to self-stigma among pregnant and parenting people with SUD [as recommended by Crandall and Holder; (83)], and using continuing education, self-evaluation tools, and professional training to reduce professional stigma. As awareness grows of the impact of stigma on individuals with SUD accessing care, the National Institutes of Health (NIH) has recently released various funding opportunities for research on trainings and tools designed to reduce stigma around SUD. Stigma is a fundamental cause of health inequities across the life course (18), and NIH continues to provide information and resources for stigma reduction in research [e.g., (62)].

Researchers can commit to participation in training opportunities designed to expose our normative judgments and to ensure that we have meaningful contact with the communities that are the focus of our research. For instance, the Mental Health First Aid training program, which focuses on mental health and substance use issues, has been shown to enhance knowledge and reduce stigmatizing perceptions (84). A growing number of training programs are available, such as those offered by Zero Block Society (85) and Prevention Technology Transfer Centers (86), as well as the perinatal harm reduction toolkit [e.g., (87, 88)]. More such training programs are needed. The NIH offers training to reduce stigma toward people who use opioids for primary care clinicians through their HEAL Initiative (88), though we are not aware of training targeted for researchers. The Center for Parenting and Opioids (CPO) has offered several trainings for researchers, including one on community-based research focused on partnerships between researchers and harm reduction frontline workers in Vancouver, British Columbia (the recording is publicly available^1^).

Contact-based training and education programs are effective for addressing stigma (89), including among medical students and professionals (34). Engagement with people who have the lived experience of substance use while pregnant and/or parenting during stigma reduction trainings may enhance the effectiveness of stigma reduction training (90). Articles that highlight the lived experiences of pregnant and parenting people with SUD can be included in stigma reduction training, such as from the perinatal harm reduction coalition. At the time of this writing, the Journal of Substance Use and Addiction Treatment offers regular “lived experience” publications designed to “honor stories of addiction and recovery, mitigate stigma through a humanized portrayal of persons, families and caregivers affected by SUD, raise issues of social justice and inequity, explore the dynamics of patient-clinician relationships, and foster compassionate engagement with people with SUD” (91) and has published qualitative reports of the lived experience of pregnant and parenting people with SUD (92).

#### Strategy #2: engage in interdisciplinary and transdisciplinary collaborations

A variety of perspectives, frameworks, and lived experiences are needed to identify and address stigmatizing elements in research dissemination. From the perspective of expanding frameworks and approaches among researchers themselves, interdisciplinary and transdisciplinary approaches have the potential to shed light on the unrealized elements of our research that may otherwise introduce stigmatizing frameworks. The lack of careful examination of information being reviewed and communication issues across fields (93) demands an increase in ongoing collaboration with individuals from different fields of study. Transdisciplinary collaborations may enhance understanding about the role of stigma in treatment outcomes and address the complex, multifaceted social phenomenon of stigma within research products [e.g., (94, 95)]. NIDA has funded several Transdisciplinary Prevention Research Centers (TPRCs) to “foster innovative translation of theories across disciplines” and “overcome the barriers inherent in integrating cross-disciplinary concepts, methods, and findings” (96).

Interdisciplinary and transdisciplinary collaborations may represent multiple benefits. For instance, they may enhance understanding of multiple streams of evidence and generate more practical and robust knowledge about the life course impacts of both fetal drug exposure and SUD-related stigma. Research collaborations also represent an opportunity for research teams to mutually inform one another’s approaches to stigma reduction. This would further promote clear and adaptable stigma reduction strategies that can be applied to many fields. Finally, collaborative approaches increase the likelihood of engaging with researchers with SUD in the context of pregnancy and parenting, who bring an informed perspective and awareness to the research process (61). While individual discretion related to self-disclosure of stigmatized identities among researchers with SUD should take precedence, doing so may further promote empowerment and reduce self-stigma (89), especially when safe spaces to do so exist.

#### Strategy #3: use community-based approaches and engage community partners

Research initiatives are too often out of touch with the communities they seek to study or serve (97). For instance, researchers may assume that parents who use substances have access to certain kinds of technology or fail to identify historical and cultural elements that inform their degree of engagement. We may not recognize misalignments between the values driving proposed studies and those of pregnant and parenting individuals with SUD (98), leading to oversights that may perpetuate stigma. Meta-analyzes and systematic reviews have demonstrated that community-based, patient-centered approaches can improve utilization and treatment outcomes for evidence-based substance use treatments (99, 100).

Community-based practices, such as engaging community advisory boards and digesting information from community surveys prior to introducing community health services, represent an ethical standard of inclusivity and have been found to reduce stigma and enhance care seeking (101). Community-based participatory research (CBPR) approaches can guide researchers in this process [e.g., having a prevention focus, being population-centered and collaborative, taking a multi-disciplinary approach, building on community strengths and resources, attending to social inequalities, taking an ecological perspective, and prioritizing mutual benefits for all partners; (102, 103)]. Researchers can increase community involvement by identifying relevant partner organizations (e.g., non-profits, community health centers, and peer organizations), planning research activities that meaningfully engage pregnant and parenting people with SUD in the research dissemination process (e.g., public presentation of study findings), ensuring that findings are communicated in understandable and usable ways (Patient-Centered Outcomes Research Institute; PCORI), and providing interventionists and practitioners structures for soliciting and communicating feedback passed along to them by participants and community partners about research products. Community partners should represent the specific population within the research, including subpopulations of pregnant and parenting people with SUD, who may differ in factors such as substances used or features of their SUD–as well as substance-specific stigmatization. For instance, not convening individuals with alcohol use disorder to provide insight into stigmatizing experiences among those with opioid use disorder who are injecting substances. This is even more relevant considering that those who use certain substances may themselves stigmatize those who use other substances. Pregnant and parenting people that use highly stigmatized substances are silenced by the systems they engage with. Researchers must understand how to create space, actively listen, and validate the experiences of these vulnerable groups. Including a member of the research team with experience facilitating groups, especially a researcher with lived experience, may improve the experiences of participants. Highly studied populations frequently emphasize the importance of researchers committing to real and meaningful benefit to study participants, including a plan for actively advocating for change based on research findings [e.g., (60)]. Community-based practices encourage the creation of valuable materials (i.e., related to childcare, lactation spaces, or parenting practices) or novel resources for participants (e.g., affinity groups for those who have experienced loss or child removal).

The Patient-Centered Outcomes Research Institute^2^ provides grant funding and an online repository of engagement-related tools and resources including “stakeholder engagement plans,” to enhance effectiveness and likelihood of translation and dissemination of research. In addition to informing our research on the whole, involvement of pregnant and parenting people with SUD ensures relevancy of the research to this community and can help bring attention to potentially stigmatizing aspects of the research. Thong et al. (104) recommends researchers receive training on building rapport and engaging more deeply with prospective participants by involving participants’ families or significant others and taking time to discuss important elements of the research process. Others point to working with peer outreach workers and organizations to help build trust and participation (105, 106).

In addition to connecting directly with pregnant and parenting people who use substances, researchers can partner with Community-Based Organizations (CBOs) who engage with this group (107). CBOs are often familiar with community strengths, needs, and challenges. PCORI’s “Stakeholders’ Substance Use Research and Treatment Information Exchange” (SSURTIE) supports efforts to design and develop infrastructure to promote increased collaboration among researchers who focus on SUD and the community partners who are affected by the disorder or who provide treatment (108). Guidelines frequently emphasize the importance of developing trusting relationships with community partners and allowing their input to define the evidence collected, critically reflecting on and dismantling power structures, exploring potential adaptations relevant to the community being considered, and engaging in critical evaluation of how scientific frameworks and approaches may contribute to barriers by perpetuating stigma.

#### Strategy #4: address stigmatizing language in science communication

Stigma toward substance use and SUD has become embedded in language itself, in part due to political rhetoric aimed at reducing substance use (109, 110). For instance, the terms “addict” and “junkie” contribute to stigma among people with SUD (111). Calls have been made for scholars to “carefully and intentionally consider the language used to describe... drug use and disorders, the individuals affected by these conditions, and their related behaviors, comorbidities, treatment, and recovery” within research products (68), pp. 2. Indeed, language influences public perceptions regarding the cause and modifiability of substance use, as well as personal perceptions related to self-efficacy among those impacted (68). As language is a known driver of social and individual perceptions of SUD, examining our language choices within research products related to pregnant and parenting individuals may reduce stigma toward this population. Synthesizing these recommendations, we propose the following strategies: use language that (1) Respects the worth and dignity of all persons (“person-first language”), (2) Focuses on the medical nature of SUD and treatment, (3) Promotes the recovery process, and (4) Avoids perpetuating negative stereotypes and biases with slang and idioms (68).

“Person-first language” emphasizes the person over their condition when referring to individuals affected by SUD, for example, “people with OUD” instead of “opioid users.” The mere use of the term ‘substance abuser’ versus ‘having a substance use disorder,’ leads to beliefs that individuals with SUD engage in willful misconduct, pose a greater threat to society, and are more deserving of punishment (112). Nonetheless, person-first language remains uncommon in the research literature on substance use (113). The research community can adopt the use of person-first language (rather than disease-first terminology) by using terms such as ‘person with a SUD’, ‘person who uses drugs’, or ‘parent who misuses opioids’ instead of ‘substance abuser’ or ‘substance using’ (19, 112). The Drug Policy Alliance recommends the use of the terms “person with substance use disorder or SUD” and “person who uses/injects drugs (PWUD/PWID)” (114). Gender specific language is important in some contexts, such as when examining differential outcomes or tailored treatment approaches. Researchers can use gender inclusive language when reporting about gender non-specific research related to pregnant and parenting people, who can be of all genders. For instance, the use of gender inclusive terms such as “pregnancy,” “pregnant people,” “during pregnancy,” “birth,” and “birthing parent.” Researchers can also practice inclusivity through conducting research with pregnant and parenting people who are not cis-gendered. In general, there is little information about the parenting experiences of men with SUD (115, 116) and far less (if anything) about people who are not cis-gender (117).

Regarding children who have been exposed to opioids *in utero* and/or have become physiologically dependent on substances they were exposed to *in utero*, the National Perinatal Association recommends the phrase child with “prenatal substance exposure” or “physiological dependance,” rather than with the term “addict” or “addicted baby,” (118). They highlight the importance of underscoring that withdrawal is a temporary and treatable condition and that their drug exposure does is not fundamentally deterministic of their long-term outcomes overall (118). This value-neutral language focuses on the effects of the use of substances, rather than the consequences, when referring to the individual.

The Associated Press Stylebook (119) provides journalistic guidelines for language use within articles related to mental illness. NIH has developed a resource that outlines various considerations when reporting scientific findings and has published a *Checklist for Communicating Science and Health Research to the Public* (120), which includes a guideline for researchers to “convey information in a respectful tone that [does not] stigmatize or assign blame to individuals or groups [affected by the disorder]” (121). The Recovery Research Institute has created the “addictionary” for the purpose of creating a unified language to destigmatize addiction (122). Several institutes, including NIDA and the National Institute on Alcohol Abuse and Alcoholism (NIAAA), have their own guidelines for person-first language. Community advisory board members can also be consulted about the terms they use when describing themselves, and the terms they prefer never to be used (123).

#### Strategy #5: provide contextualizing information about the social and environmental factors that influence substance use among pregnant and parenting people

SUD, pregnancy, and parenting do not occur independently of social, environmental, and genetic factors, which have all been demonstrated as determinants of substance use initiation (124). Social and structural determinants of substance use, such as socioeconomic status, disparities in healthcare delivery, discrimination, racism, and social exclusion, have been well-documented (25, 125, 126). These structural factors, along with fear of related stigma, additionally drive treatment engagement (14). Without critically examining their frameworks and analytical approaches, researchers may unintentionally over-emphasize the role of parental substance use in public health issues, neglect to consider how the framing of study results will be perceived by society, or engage in “stigma by omission” (i.e., failing to acknowledge the important role of social and structural factors in SUD). Researchers can work to avoid the tendency to discount social contextual factors that influence child development and behavioral changes, which are more robust predictors of these outcomes than substance exposure alone (127). As misconceptions about the nature and strength of the relationship between exposure and developmental outcomes (see 10) may lead to researcher prejudice, addressing these misconceptions is needed. Despite extensive evidence of contextual and genetic influences on substance use, theoretical frameworks presented in scientific communications often do not acknowledge the role of factors outside individuals. This kind of de-contextualized communication may overemphasize the role of individual characteristics, such as lack of motivation or interest on the part of the individual, as the primary drivers of substance use and treatment non-initiation. For instance, many scientific frameworks for understanding substance use exclusively focus on internal psychological and behavioral mechanisms such as self-regulatory processes and habit extinction (128, 129). While individual-level factors are certainly related to substance use disorder trajectories, a failure to nest individual factors within the broader context may influence self-stigma related to personal ability to recover (130) and stigmatization within treatment systems of care (8). This lack of context may even translate to our statistical approaches. As noted by Terplan (131), p. 1729:

‘Categories such as race, gender, pregnancy, poverty, immigration status, sexual orientation, and medical comorbidities interact in an integrative (not additive or multiplicative) fashion. Logistic regression, even when augmented by sensitivity analyses, therefore executes a leveling effect on the data and, with each turn of the model, whittles away the richness, nuance, and suffering that is the human experience.’

In this way, guiding theories and patterns of data interpretation often “fail in scope to represent the many experiences of addiction” (61), p. 2.

Researchers can reduce stigma toward pregnant and parenting people who use substances by ensuring accurate representation of existing research and considering whether previous research has appropriately accounted for contextual factors. This includes exercising caution not to overstate findings. Researchers can vet research that is peer-reviewed and evidence-based, such as high-quality observational studies that adjust for potential confounding, randomized controlled trials, or meta-analysis. Adjusting for confounding (e.g., of socioeconomic and sociocultural variables) is especially important regarding reporting of biological effects on *in-utero* exposure to substances and when studying parenting behaviors, which are often sensationalized.

Contextual theoretical frameworks such as Bronfenbrenner’s Social Ecological Framework (132) or Engel’s Biopsychosocial Model (133) outline the multiply determined nature of SUD. Contextual frameworks incorporate key drivers in the development and onset of SUD, such as social isolation or social rejection [see (116, 127) for examples]. By contextualizing substance use within research frameworks, researchers can frame the biological, social, and structural drivers of substance use. These frameworks may both address the determinants driving substance use more effectively and shift public perceptions regarding those who are affected by SUD. This can be done even if the study did not take a contextual approach during the design phase, through providing this context in the introductory and discussion sections of the paper.

Research related to the impact of substance use on pregnant and parenting people and their families often focuses heavily on the outcomes related to children [e.g., (134)], rather than providing a balanced lens that also considers the parents’ wellbeing and the functioning of the family. This has led, for instance, to a research base that minimizes the harmful effects of the removal of children from their families. Family preservation approaches emphasize the responsibility of society to minimize the destructive potential impact of the child welfare system (135). Further, research products that overly focus on the child may inadvertently generate additional stigma toward children whose parents use substances. Indeed, people who experienced parental substance use report experiences of stigma and social exclusion (136). Research efforts aimed at supporting families in the context of substance use can consider how the parent might be best supported while they are with their child(ren).

#### Strategy #6: advocate for stigma-reducing policies in research articles and other scholarly products

Research plays a role in creating and amending policy. This occurs directly through channels by which research is communicated to policymakers and indirectly through influencing public interpretation of research theories and results (137). For instance, research informs evidence-based policies that have downstream impacts on treatment of parental SUD, including within the child welfare system (138). Indeed, research on the importance of parent–child relationships has encouraged policies that reunite children who have been removed from their home as a result of substance use, as opposed to previous policy that promoted children remaining with temporary caregivers (138). Careful evaluation of how we are portraying the implications of our findings may prevent unintended contribution to harmful policy.

Researchers can discuss our findings and their implications within the broader context of social policy in ways that minimize stigma. Grantmaking bodies are increasingly inviting researchers to submit “impact statements,” which are intended to demonstrate the potential benefit research might have in the world. Impact statements that (1) plainly discuss how findings relate to family policy and (2) clearly address how findings could be misinterpreted to promote stigmatizing family policy are critical for stigma reduction. Impact statements are an opportunity to clearly address what findings do and do not imply for public policy. For instance, researchers who evaluate the effects of substances on parenting practices can emphasize that their individual findings do not imply support for unethical child removal practices when applicable. Researchers can also make connections from their findings to policies that reduce harm for parents with SUD and their children (i.e., reunification practices and social support services).

Researchers can also proactively advocate for the development of frameworks and guidelines for stigma reduction when conducting research from influential regulatory and grantmaking bodies. Locally, researchers can work with their Institutional Review Boards (IRBs) to advocate for frameworks and guidelines for stigma reduction when conducting studies. IRBs can be encouraged to develop guidelines related to non-stigmatizing terminology and to support researchers to meaningfully consider practices to reduce stigma resulting from research participation for pregnant and parenting individuals who use substances. Nationally, laws related to stigma-reduction practices would afford dissemination guidelines a judicial basis and ensure that ethical review boards work to uphold these ethical standards (139). Researchers can also directly engage in dissemination efforts that connect our research to policies which impact pregnant and parenting individuals with SUD and their families. For instance, becoming aware of gaps in policy can provide insight into where more research is needed. The Research-to-Policy Collaboration (140) is one organization that connects researchers to relevant needs in policymaking. While research can directly inform policy, we can actively use our leverage as researchers to inform public opinion, including through op-ed papers. Publishing expert opinion in public news draws the attention of policy makers and promotes social pressure that can aid in the development or amendment of policy.

See Table 2 for a summary of proposed strategies and actionable recommendations.

**Table 2:** Actionable steps to reduce stigma within research dissemination.

Strategy	Key message	Actions researchers can take
1. Address Researcher Stereotypes, Prejudice, and Misconceptions about Pregnant and Parenting People with SUD	Judgments, biases, and misconceptions on the researcher’s part may influence public and self-stigma, addressing these is critical.	Participate in trainings to expose normative judgments and engage with pregnant and parenting people who experience SUD (e.g., Mental Health First Aid, Zero Block Society, or Prevention Technology Transfer Centers)
		Read The Journal of Substance Use and Addiction Treatment “lived experience” publication series and harm reduction toolkits (e.g., from the Perinatal Harm Reduction Coalition).
2. Engage in interdisciplinary and transdisciplinary collaborations	Transdisciplinary collaborations help us to understand and address stigma, which is a complex, multilevel social phenomenon. Researchers with lived experience exist and have valuable insights and can provide valuable insight into how to reduce stigma in research products for dissemination. They can also act as brokers between other researchers and the community.	Invite researchers from relevant fields to support the framing within research products.
		Engage with researchers who are abstinent/in recovery or currently use drugs to consult on the language used in scholarly products.
		Create collaborative spaces for researchers with and without lived experience to engage with community partners on language and dissemination practices.
		Follow work from NIDA’s Transdisciplinary Prevention Research Centers (TPRCs)
3. Use community-based approaches and engage community partners	Research approaches that actively engage the community partners most involved with and impacted by the conditions being researched (e.g., community-based research models) may successfully reduce stigma.	Conduct community advisory boards.
		Access PCORI’s resources (e.g., “stakeholder engagement plans”).
4. Address stigmatizing language in science communication	Community-informed resources for de-stigmatized terms (e.g., person-first language) are available and constantly evolving.	Avoid the use of slang and idioms.
		Use current resources, such as NIDA’s “words matter” resource, “the addictionary,” and NIH’s recommendations for reducing alcohol-related stigma that inform the use of de-stigmatized terminology.
		Focus on the medical nature of SUD and treatment.
		Use “recovery-promoting” language.
5. Provide contextualizing information about the social and environmental factors that influence substance use among pregnant and parenting people	Theoretical frameworks presented by the researchers shape self and public perception - utilizing contextual frameworks that recognize the multiply determined nature of substance use may minimize stigma.	Utilize contextual theoretical frameworks that acknowledge biological, social, political, and environmental determinants of SUD within research products.
		Ensure accurate representation of existing research
		Avoid overly focusing on the child
6. Advocate for stigma-reducing policies in research articles and other scholarly products	Policies and guidelines reinforce scientific procedures and communication strategies, which can be leveraged to reduce stigma.	Clearly state how public policy is affecting your sample in academic papers.
		Write to funding and publishing bodies to advocate for the development of stigma-reducing policies and guidelines.
		Learn about the research-to-policy collaboration

#### A case study: stigma-reduction efforts at the center on parenting and opioids

While implementing these practices is a long-term goal that is not without barriers, we aim to support researchers in taking steps toward minimizing substance-related stigma. The authors of this publication are colleagues at The Center on Parenting and Opioids (CPO), where we are implementing steps to align with the strategies outlined in this paper. The CPO is funded by NIDA and is jointly housed at the University of Oregon and Oregon Health & Science University. We hope our work at the CPO provides an example of taking meaningful steps toward bridging the gap between rigorous science and community impact. From professional workshops to a Knowledge Dissemination working group, the CPO’s initiatives seek to not only challenge researchers to engage reflexively with their research, but to also guide science communicators toward sharing research outcomes and policy recommendations in a way that reduces stigma and expands accessibility to information.

One such initiative is the composition of research briefs that synthesize key findings from current CPO-affiliated studies, with a focus on highlighting the practical utility of science. To actively work toward reducing the stigmatizing language that all too often surrounds research on substance use–particularly regarding pregnant and parenting people who use substances–the CPO has also generated a *Destigmatizing Research Dissemination Checklist* specifically intended for those writing and reviewing the research briefs. The *Destigmatizing Research Dissemination Checklist* includes items such as “building on community strengths” and “highlighting the utility of research,” with practical tips like (1) writing with a collective tone to avoid othering the communities we seek to positively impact and (2) using destigmatizing and person-first language. While attending to language and terminology is just one step toward reducing stigma in science (see Table 2), the CPO’s research briefs and Checklist represent an initiative designed to prioritize stigma reduction in research products. Our aim is for these institutional changes to impact social, self, and structural stigma.

## Discussion

The goal of this set of strategies is to empower researchers to destigmatize their research products. We anticipate that by reducing stigma in research products, we will be able to reduce self-stigma, social stigma enacted by professionals who care for pregnant and parenting people with SUD, and structural stigma upheld by organizational, local, state, and federal policies. However, there are limitations to our proposed strategies. First, we understand that it takes time and energy to engage with community partners. While we believe their investment and direction is well worth the additional expenditures, we understand that community-engaged research is a new skill that many researchers do not currently practice or have access to training opportunities to learn. We also understand that the academic cycle does not currently well-incentivize community engagement, but incentivizes the quantity of scholarly products published in peer-reviewed journals and grants that bring institutional funding. Thus, destigmatizing research products also requires that the approaches outlined in our work are institutional priorities.

This paper explores opportunities to minimize stigma in the dissemination of research findings to relevant clinical, regulatory, and community audiences, with a specific focus on pregnant and parenting individuals with SUD. However, stigma reduction efforts are needed at all levels of the research process (e.g., planning, research conduct, and interpretation of findings). Examinations of stigma reduction practices across research phases is needed to produce research that accurately and substantively represents those individuals with SUD (141). Enacted stigma at every phase of research can create barriers to participation and preclude many people with SUD from feeling able or being able to participate in studies, ultimately limiting the generalizability of research findings. Additional work is needed to address participation barriers not discussed extensively here, including physical resource limitations [e.g., unstable housing, limited access to transportation, or lack of childcare; (111)], as well as related barriers, such as generalized mistrust of researchers, fear of legal repercussions, or duration and magnitude of the study (142).

The stigma reduction framework outlined here can be used to guide the examination of ways in which stigma has been perpetuated in prior research products. We also suggest that our proposed strategies may be modified to consider how to reduce stigmatization during other research processes.

We have argued that low participation in evidence-based substance misuse prevention and harm reduction programs among pregnant and parenting people who use substances is due in part to the role of stigma toward this group. We emphasize the detrimental impact of stigma at the level of the individual, society, and institution which includes undermining efforts to prevent and treat SUD and perpetuates health inequities. We examined the role of research in perpetuating stigma and its downstream effects and provided guidance based on available theory and evidence for practices to reduce stigma within our research products. These are: (1) address researcher stereotypes, prejudice, and misconceptions about pregnant and parenting people with SUD; (2) engage in interdisciplinary and transdisciplinary collaborations - emphasizing engagement with researchers with lived experience; (3) use community-based approaches and engage community partners, (4) address stigmatizing language in science communication; (5) provide context for research related to pregnant and parenting people with SUD; and (6) advocate for stigma-reducing policies in research articles and other scholarly products. By endeavoring to reduce stigma in our research dissemination practices, we may not only improve our theories, but also the degree to which our findings have real-world impact that minimize stigmatization and discrimination and ultimately transform health outcomes for families impacted by SUD.

## Author contributions

ML, SM, KW-S, and CC contributed to conception of the paper. ML was the primary author of the manuscript. KW-S, SM, EB, and CC wrote sections of the manuscript. CC, ML, ETB, and MT provided edits and feedback to the draft. ML and CC revised the manuscript. All authors read and approved the submitted version.

## Funding

This work was supported by the National Institute on Drug Abuse of the National Institutes of Health under Award Number P50DA048756 (PIs Leve & Fisher). The content is solely the responsibility of the authors and does not necessarily represent the official views of the National Institutes of Health. ML and ETB were supported by grants CA240452 and CA21122.

## Conflict of interest

The authors declare that the research was conducted in the absence of any commercial or financial relationships that could be construed as a potential conflict of interest.

## Publisher’s note

All claims expressed in this article are solely those of the authors and do not necessarily represent those of their affiliated organizations, or those of the publisher, the editors and the reviewers. Any product that may be evaluated in this article, or claim that may be made by its manufacturer, is not guaranteed or endorsed by the publisher.

## Footnotes

^1^ https://www.cpo.uoregon.edu

^2^ https://www.pcori.org/
